# Falls resulting in health care among older people with intellectual disability in comparison with the general population

**DOI:** 10.1111/jir.12564

**Published:** 2018-11-08

**Authors:** A. Axmon, G. Ahlström, M. Sandberg

**Affiliations:** ^1^ Division of Occupational and Environmental Medicine, Department of Laboratory Medicine, Faculty of Medicine Lund University Lund Sweden; ^2^ Department of Health Sciences, Faculty of Medicine Lund University Lund Sweden

**Keywords:** accidental falls, fractures, health services research, intellectual disability, older adults, wounds and injuries

## Abstract

**Background:**

Falls are common among older people with intellectual disability (ID) and are also a major contributor to injuries in this population. Yet, fall characteristics have only been sparsely studied, and the results are inconsistent. The aim of the present study was to investigate type of falls, places where they occurred and activities that caused them, as well as health outcomes and health utilisation patterns after falls, among older people with ID in comparison with their age peers in the general population.

**Methods:**

We established an administrative cohort of people with ID aged 55 years, or more, and alive at the end of 2012 (ID cohort; *n* = 7936). A cohort from the general population, one‐to‐one matched by sex and year of birth, was used as referents. Data regarding fall‐induced health care episodes in inpatient and outpatient specialist care were collected from the National Patient Register for the period 2002–2012.

**Results:**

With the exception of falls from one level to another (i.e. fall on and from stairs and steps, ladder and scaffolding; fall from, out of or through building or structure; fall from tree or cliff and diving or jumping into water; or other fall from one level to another), people in the ID cohort were more likely to fall and fall more often than those in the general population cohort. Falls during a vital activity (e.g. attending to personal hygiene or eating) were twice as common among people with ID compared with the general population. When falling, people with ID were more likely to injure their head and legs but less likely to sustain injuries to the thorax and elbow/forearm. They were more likely to have superficial injuries, open wounds and fractures but less likely to have dislocations, sprain and strains. Fall‐related health care visits among people with ID were more likely to be in inpatient care and be unplanned. People with ID were also more likely than those in the general population to have a readmission within 30 days.

**Conclusions:**

People with ID are more likely to require specialist care after a fall and also more likely to obtain injuries to the head, compared with the general population. This is important to consider when taking preventive measures to reduce falls and fall‐related injuries.

## Background

Falls are common among people with intellectual disability (ID) (Sherrard *et al*. 2001; Cox *et al*. 2010; Finlayson *et al*. 2010; Hsieh *et al*. 2012; Smulders *et al*. 2013; Enkelaar *et al*. 2013a; Foran *et al*. 2016; Sandberg *et al*. 2017; Finlayson 2018). In a recent review, Finlayson (2018) described how falling may have serious consequences for people with ID, including loss of confidence, injury and death. Indeed, falling is a major contributor to injuries among people with ID (Hsieh *et al*. 2001; Finlayson *et al*. 2010). Injuries, in turn, are a major cause of hospitalisation (Skorpen *et al*. 2016). However, although people with ID are equally likely as people in the general population to be admitted to hospital for injuries, they are less likely to have injury‐related emergency room visits (Wang *et al*. 2002). Thus, in order to enable preventive measures and ensure proper health care after falls in this vulnerable population, it is important to investigate the characteristics of their falls, as well as current health care patterns associated with falls among them. However, the scarce data regarding fall characteristics among people with ID are inconsistent. For example, whereas one study (Pal *et al*. 2014) found that most falls occurred indoors, another (Smulders *et al*. 2013) found outdoor falls to be more common. A qualitative study by Cahill *et al*. (2014) identified 17 different themes that contributed to falls and that could be organised under three headings: individual, behavioural or contextual factors. This study also found that falls affected both the individual with ID, in terms of direct consequences as anxiety, and reduced independence, as well as their carer, with feelings of guilt and support demands. In addition, the person with ID and their carer also made several adaptions after a fall. Depending on the individual's level of insight, this included changed mobility patterns, increased reliance on external support among people with ID, modifications to the environment and greater physical and verbal assistance among the carers (Cahill *et al*. 2014).

In recent decades, the life expectancy of people with ID has increased (Coppus 2013), as has the proportion of people with ID aged 65 years, or more (Bigby 2007; Blackman 2007). In the general population, increasing age is a major risk factor for falls (World Health Organization 2007). Even so, of the studies described earlier, only a few have focused on older people with ID (Smulders *et al*. 2013; Enkelaar *et al*. 2013a; Foran *et al*. 2016; Sandberg *et al*. 2017), and only one included comparisons with a same‐aged group from the general population (Sandberg *et al*. 2017), and then only to compare occurrence of falls. Thus, to the best of our knowledge, there is a lack of literature regarding fall characteristics and fall‐related health care needs among older people with ID in comparison with the general population.

The aim of the present study was to investigate type of falls, places where they occurred and activities that caused them, as well as health outcomes and health care utilisations patterns after falls among older people with ID in comparison with their age peers in the general population.

## Methods

The present study is register based, using Swedish national registers both to identify the study cohorts and to provide information on outcomes (i.e. falls).

### Cohorts

A cohort of people with ID (ID cohort) was established based on the so‐called LSS register. This register is maintained by the Swedish National Board of Health and Welfare and contains national data on support and services provided to people with ID or autism spectrum disorder (ASD) according to the Act Concerning Support and Service for Persons with Certain Functional Impairments (the LSS act) (SFS 1993:387, 1993). All people aged 55 years, or more, with at least one measure of support during 2012, and alive at the end of this year were included in the cohort. We chose 55 years as cut‐off as ageing occur at an earlier chronological stage among people with ID (Haveman *et al*. 2010). Although a diagnosis of either ID or ASD is required to obtain support according to the LSS act, no diagnosis is recorded in the register. Thus, we may have included people with ASD but without ID.

Statistics Sweden provided a referent cohort of people from the general population (gPop), matched by sex and year of birth. Each cohort comprised 7936 people (3609 women and 4327 men) aged 55 years, or more, and alive at the end of 2012.

### Outcomes

The Swedish National Patient Register contains information on all inpatient care episodes and outpatient specialist visits. When the health care episode is caused by a particular reason, as defined by the ICD‐10 chapter XX (external causes for morbidity and mortality), the physician should record this external cause as well as a primary diagnosis. For example, if a person falls from a tree and fractures his or her arm, the primary diagnosis would be recorded as fracture of shoulder and upper arm (S42), and the external cause would be registered as fall from tree (W14). We defined fall‐related health care contacts as those registered with W00–W19 during the study period (2002–2012) and used these as a proxy for falls.

We investigated three groups of outcomes: fall characteristics, health outcomes after falls and patterns of health care utilisation due to falls.

#### Fall characteristics

To describe fall characteristics, we used number of falls during the study period, type of fall, place where the fall occurred and the activity the person was engaged in at the time of fall.

Information on type of fall was obtained from the ICD‐10 codes for the health care visits and aggregated to *falls on the same level* [fall on the same level involving ice and snow (W00), fall on same level from slipping, tripping and stumbling (W01), fall involving ice‐skates, skis, roller‐skates or skateboards (W02), other fall on same level due to collision with, or pushing by, another person (W03) and other fall on same level (W18)], *falls involving furniture/equipment* [fall involving wheelchair (W05), fall involving bed (W06), fall involving chair (W07), fall involving other furniture (W08) and fall involving playground equipment (W09)], *falls from one level to another* [fall on and from stairs and steps (W10), fall on and from ladder (W11), fall on and from scaffolding (W12), fall from, out of or through building or structure (W13), fall from tree (W14), fall from cliff (W15), diving or jumping into water causing injury other than drowning or submersion (W16) and other fall from one level to another (W17)] and *other falls* [fall while being carried or supported by other persons (W04) and unspecified fall (W19)].

The fourth digit of the ICD‐10 code was used to determine the place of the fall, specified as home, institution, school or other public area, sport facility, street, shopping and service area, industrial area, agricultural area or other area. The fifth digit of the ICD‐10 code was used to determine the activity when the fall occurred, specified as sports, hobby or free time, work, other occupation, vital activity, other activity or unspecified activity.

#### Health outcomes after falls

We assessed health outcomes as primary diagnosis for the visit, injured body part and injury type. When the primary diagnosis was injury to a specific body part, information on injured body part was collected from the second digit of the ICD‐10 code. Available options were head, neck, abdomen/lower back/lumbar spine/pelvis, shoulder/upper arm, elbow/forearm, wrist/hand, hip/thigh, knee/lower leg and ankle/foot. Moreover, when the primary diagnosis was injury to a specific body part, information concerning the type of injury was obtained from the third digit of the ICD‐10 code. Available options were superficial injury, open wound, fracture, dislocation, sprain/strain, traumatic amputation, and other injuries.

#### Patterns of health care utilisation due to falls

In order to study patterns of health care utilisation, we collected information on type of care, length of stay and readmissions. When considering type of care, we categorised each visit as either planned (i.e. when the appointment was made beforehand) or unplanned, and determined if the visit was to inpatient or outpatient specialist care, and to which clinic. Length of stay for inpatient care episodes was determined for each individual, both as the total time during the study period and as the average time for each inpatient care episode. Readmissions were defined as a second fall‐related health care contact within 30 days.

### Statistics

Analyses of dichotomous outcomes (e.g. having at least one fall) were performed using generalised linear models, estimating relative risks with 95% confidence intervals. Categorical outcomes with more than two levels were analysed using Pearson's chi‐square. Number of falls was compared using Mann–Whitney *U*‐test.

All analyses were performed using IBM SPSS Statistics version 23.0. Analyses were only performed when both groups to be compared comprised more than five individuals. A two‐sided *P*‐value below 0.05 was considered statistically significant.

### Ethics approval

Approval was obtained from the Regional Ethical Review Board in Lund (no. 2013/15). The National Board of Health and Welfare and Statistics Sweden performed separate secrecy reviews before providing access to the data. All analyses were performed using anonymised data sets. The authors assert that all procedures contributing to this work comply with the ethical standards of the relevant national and institutional committees on human experimentation and with the Helsinki Declaration of 1975, as revised in 2008.

## Results

### Fall risk

**Figure 1:**
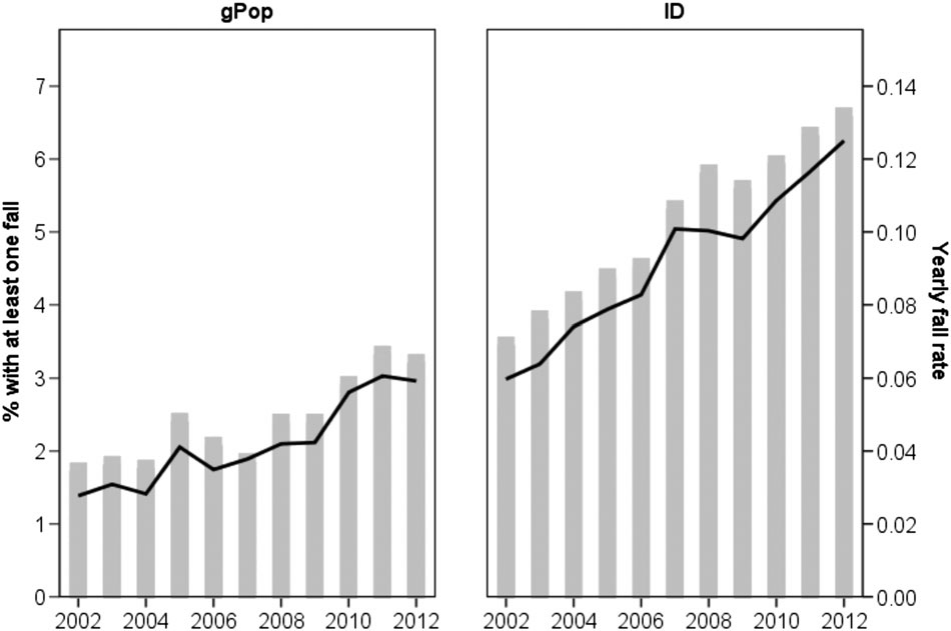
Yearly percentages of people with at least one fall (bars) and yearly fall rate (line) in a cohort of people with intellectual disability (ID) and a referent cohort from the general population (gPop), one‐to‐one matched by sex and year of birth.

The yearly incidence of falls increased over time in both cohorts, from 3.5% in 2002 to 6.6% in 2012 in the ID cohort and from 1.7% to 3.2% in the gPop cohort (Fig. 1). Similarly, the yearly fall rate increased from 0.06 to 0.13 in the ID cohort and from 0.03 to 0.06 in the gPop cohort.

**Figure 2:**
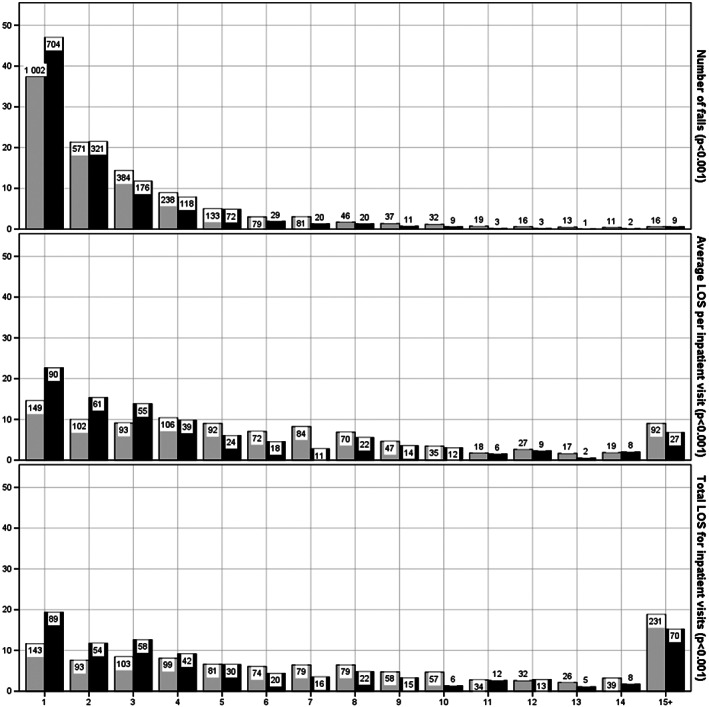
Percentage (*y* axis) and number (data labels) of people with intellectual disability (grey) and in a general population sample (black) with different number of falls and length of stay (LOS) due to falls during the study period.

People in the ID cohort had increased risk of all types of falls except for falls from one level to another (Table 1). Among those with at least one fall, the median number of falls was two. This was true in both cohorts as well as in all subgroups within the ID cohort. However, there was a shift towards more falls (i.e. a longer ‘tail’ of the distribution above the median) in the ID cohort compared with the gPop cohort (*P* < 0.001; Fig. 2).

**Table 1:** Falls leading to health care contacts among 7936 people with intellectual disability (ID) and a same‐sized sample from the general population (gPop) matched by sex and year of birth

Outcome according to ICD‐10 code	Falls		Individuals		
	gPop	ID	gPop	ID	ID vs. gPop
	*n* (%)†	*n* (%)†	*n* (%)‡	*n* (%)‡	RR (95% CI)
Any fall	3657	8005	1498 (19)	2678 (34)	**1.79 (1.69–1.89)**
On the same level	2083 (57)	4517 (56)	985 (12)	1941 (24)	**2.03 (1.89–2.18)**
Involving furniture/equipment	50 (1)	465 (6)	39 (0)	251 (3)	**6.44 (4.60–9.01)**
From one level to another	478 (13)	311 (4)	247 (3)	167 (2)	**0.68 (0.56–0.82)**
Other	1046 (29)	2712 (34)	587 (7)	1221 (15)	**2.08 (1.90–2.28)**
Place of fall					
Home	667 (18)	1249 (16)	375 (25)	744 (28)	1.11 (1.00–1.24)
Institution	38 (1)	989 (12)	23 (2)	569 (21)	**13.8 (9.17–20.9)**
School, public area	32 (1)	68 (1)	21 (1)	39 (1)	1.04 (0.61–1.76)
Gym	56 (2)	21 (0)	36 (2)	13 (0)	**0.20 (0.11–0.38)**
Street	151 (4)	251 (3)	84 (6)	163 (6)	1.09 (0.84–1.40)
Service area	24 (1)	42 (1)	12 (1)	27 (1)	1.26 (0.64–2.48)
Industrial area	18 (0)	9 (0)	12 (1)	7 (0)	**0.33 (0.13–0.83)**
Agricultural area	16 (0)	10 (0)	13 (1)	7 (0)	**0.30 (0.12–0.75)**
Other area	83 (2)	90 (1)	50 (3)	59 (2)	**0.66 (0.46–0.96)**
Unspecified area	2572 (70)	5268 (66)	1170 (78)	2045 (77)	0.98 (0.95–1.01)
Activity when falling					
Sports	167 (5)	221 (3)	111 (7)	171 (6)	0.86 (0.68–1.09)
Hobby, free time	142 (4)	262 (3)	100 (7)	165 (6)	0.92 (0.73–1.17)
Work	88 (2)	51 (1)	51 (3)	35 (1)	**0.38 (0.25–0.59)**
Other occupation	73 (2)	78 (1)	52 (3)	60 (2)	**0.65 (0.45–0.93)**
Vital activity	150 (4)	554 (7)	86 (6)	315 (12)	**2.05 (1.63–2.58)**
Other activity	90 (2)	185 (2)	53 (4)	123 (5)	1.30 (0.95–1.78)
Unspecified activity	2946 (81)	6644 (83)	1272 (85)	2377 (89)	**1.05 (1.02–1.07)**

† Percentage based on total number of falls.

‡ Percentage based on number of people in the cohort with at least one fall during the study period.

Bold text indicates statistically significant differences. CI, confidence interval; RR, relative risk.

### Fall characteristics

Information on where the fall occurred was available for 5268 falls (66%) in the ID cohort and 2572 falls (70%) in the gPop cohort. People with ID who had at least one fall during the study period were more likely than their counterparts in the gPop cohort to have fallen at an institution (Table 1). They were less likely to have fallen in a gym or an industrial or agricultural area, as well as in ‘other areas’.

The activity at the time of the fall was recorded for 7995 (99%) of the falls in the ID cohort and for 3656 (99%) of the falls in the gPop cohort. People in the ID cohort were more likely to have fallen during a vital activity (e.g. attending to personal hygiene, resting, sleeping or eating) but less likely to have fallen during work or other occupation (Table 1). They were also more likely to have ‘unspecified activity’ recorded.

### Health outcomes after falls

The most common single primary diagnoses after fall in the ID cohort were fracture of lower leg (including ankle; S82 in ICD‐10; representing 16%, *n* = 1413 of all falls), fracture of femur (S72; 11%, *n* = 995) and fracture of forearm (S52; 9%, *n* = 795). In the gPop cohort, the most common diagnoses were fracture of forearm (S52; 16%, *n* = 596), fracture of lower leg (including ankle; S82; 12%, *n* = 426) and fracture of femur (S72; 6%, *n* = 204).

The majority of the falls in both the ID (*n* = 7219, 90%) and gPop (*n* = 3270, 89%) cohorts had a diagnosis of injured body part (chapter S in ICD‐10). Knee and lower leg was the most commonly injured body part in both cohorts, albeit tied with elbow and forearm in the gPop cohort (Table 2). People in the ID were more likely than those in the gPop cohort to injure their head or leg (hip/thigh as well as knee/lower leg) when falling. They were less likely to injure the thorax, elbow and forearm.

**Table 2:** Single body part injuries after falls leading to health care contacts among 7936 people with intellectual disability (ID) and a same‐sized sample from the general population (gPop) matched by sex and year of birth

Outcome according to ICD‐10 code	Falls		Individuals		
	gPop	ID	gPop	ID	ID vs. gPop
	*n* (%)†	*n* (%)†	*n* (%)‡	*n* (%)‡	RR (95% CI)
Body part injured					
Head	374 (11)	1080 (15)	228 (15)	668 (25)	**1.63 (1.42–1.87)**
Neck	38 (1)	62 (1)	23 (2)	29 (1)	0.70 (0.41–1.21)
Thorax	123 (4)	131 (2)	96 (6)	100 (4)	**0.58 (0.44–0.76)**
Abdomen, lower back, lumbar spine and pelvis	113 (3)	150 (2)	75 (5)	116 (4)	0.86 (0.65–1.14)
Shoulders and upper arm	461 (14)	877 (12)	221 (15)	443 (17)	1.12 (0.96–1.29)
Elbow and forearm	647 (20)	898 (12)	287 (19)	420 (16)	**0.81 (0.71–0.93)**
Wrist and hand	349 (11)	678 (9)	198 (13)	367 (14)	1.03 (0.88–1.21)
Hip and thigh	294 (9)	1205 (17)	163 (11)	664 (25)	**2.27 (1.93–2.66)**
Knee and lower leg	662 (20)	1737 (24)	323 (22)	707 (27)	**1.22 (1.09–1.37)**
Ankle and foot	209 (6)	401 (6)	159 (11)	295 (11)	1.03 (0.86–1.24)
Type of injury					
Superficial injury	377 (12)	985 (14)	300 (20)	717 (27)	**1.34 (1.19–1.51)**
Open wound	162 (5)	592 (8)	126 (8)	403 (15)	**1.79 (1.48–2.16)**
Fracture	1949 (60)	4698 (65)	831 (56)	1743 (65)	**1.17 (1.11–1.24)**
Dislocation, sprain and strain	399 (12)	536 (7)	279 (19)	365 (14)	**0.73 (0.64–0.84)**
Traumatic amputation	<5	0 (0)	<5	0 (0)	NC
Other or unspecified injury	382 (12)	408 (6)	197 (13)	293 (11)	**0.83 (0.70–0.99)**

† Percentage based on total number of falls.

‡ Percentage based on number of people in the cohort with at least one fall during the study period.

Bold text indicates statistically significant differences. CI, confidence interval; RR, relative risk. NC = not calculated due to too few observations.

The most common type of injury was fractures, which represented more than half of the injuries in both cohorts (Table 2). When falling, people in the ID cohort were more likely than their counterparts in the gPop cohort to sustain superficial injury, open wound and fracture. However, dislocation, sprain, and strain, and other/unspecified type of injury were more common in the gPop cohort.

### Health care utilisation patterns

Falls in the ID cohort were more likely than those in the gPop cohort to be recorded in inpatient care, and among those with at least one fall, people in the ID cohort were more likely to have had at least one fall leading to inpatient care (Table 3). Falls in the ID cohort were more likely than those in the gPop cohort to result in unplanned health care (inpatient and outpatient combined), and people in the ID cohort with at least one fall were more likely to have had an unplanned health care visit.

**Table 3:** Health care utilisation after falls leading to health care contacts among 7936 people with intellectual disability (ID) and a same‐sized sample from the general population (gPop) matched by sex and year of birth

	Falls		Individuals		
	gPop	ID	gPop	ID	ID vs. gPop
	*n* (%)†	*n* (%)†	*n* (%)‡	*n* (%)‡	RR (95% CI)
Type of care					
Inpatient care (planned and unplanned)	692 (19)	1889 (24)	494 (33)	1254 (47)	**1.42 (1.31–1.54)**
Unplanned care (inpatient and outpatient care)	1939 (54)	4764 (61)	1259 (86)	2422 (92)	**1.08 (1.05–1.10)**
Department visited					
Emergency	501 (14)	1134 (14)	385 (26)	669 (25)	0.97 (0.87–1.08)
Surgical	478 (13)	1109 (14)	316 (21)	666 (25)	**1.18 (1.05–1.33)**
Orthopaedic	2250 (62)	4850 (61)	970 (65)	1814 (68)	1.05 (1.00–1.10)
Other	428 (12)	912 (11)	268 (18)	630 (24)	**1.32 (1.16–1.50)**
Readmission	NA	NA	541 (36)	1066 (40)	**1.10 (1.02–1.20)**

† Percentage based on total number of falls.

‡ Percentage based on number of people in the cohort with at least one fall during the study period.

Bold text indicates statistically significant differences. CI, confidence interval; RR, relative risk.

Visits to an orthopaedic department were most common in both the ID and gPop cohorts (Table 3). Among those with at least one fall, people in the ID cohort were more likely than those in the gPop cohort to ever have their fall recorded in a surgical or ‘other’ department, whereas no differences were found for visits to the emergency or orthopaedic departments.

People in the ID cohort with at least one inpatient care episode had longer length of stay than their counterparts in the gPop cohort, both regarding per health care contact and total for the study period (both *P* < 0.001; Fig. 2). People in the ID cohort were also more likely to be readmitted within 30 days after a fall (Table 3).

## Discussion

Older people with ID are at higher risk of falls than their age peers in the general population. They are especially likely to fall at their residence and during a vital activity. When falling, they are more likely to injure their head, hips and legs. Moreover, they are more likely to have serious injuries, such as open wound and fractures, but they also have an increased risk of superficial injuries.

There are some potential weaknesses with the present study that need to be considered before interpreting the results. We used receiving support intended for people with ID or ASD as a proxy for having ID. This may have caused misclassification in two ways. First, people with ASD but without ID may have been included in the ID cohort. Both ASD and ID are diagnoses normally made during childhood and adolescence, and considering the age group investigated, we cannot expect to find these diagnoses for all study participants in the patient register during the study period. However, we found either ID or ASD diagnosis for 2494 (31%) people in the ID cohort. Of these, only 347 (14%) had ASD diagnosis but no ID diagnosis. Thus, the impact of including people with ASD but not ID should be minor. The second way would be if people with ID did not receive support and therefore were not included in the register. However, considering the age group investigated, it is unlikely that support, or sufficient support, is provided by parents. Moreover, there is no tradition in Sweden of taking care of siblings or other relatives with disabilities. Thus, it is likely that the ID cohort composes – if not all so at least a vast majority – of older people with ID in Sweden.

Both the World Health Organization and the United Nations define ‘older persons’ as those being 60 years or over. However, among people with ID, the ageing process is believed to start earlier, as indicated by earlier onset of age‐related diseases (Kapell *et al*. 1998; Janicki *et al*. 1999; Haveman *et al*. 2011; Evenhuis *et al*. 2012; Axmon *et al*. 2016). Thus, we decided to use a lower cut‐off in our definition of ‘older’ and included people 55+ years. Still, in using retrospective data, the actual age of the people in the study at the start of the study period was 44+ years. This way, we expect to have captured not only falls due to old age but also those occurring in earlier phases of the ageing process.

In the present study, we used health care visits in inpatient and outpatient specialist care registered as due to falls as proxies for severe falls, as primary care data are not included in the patient register. Thus, differences between people with ID and the general population may occur if there are differences between these two groups with respect to the prevalence of such falls but also if they result in different health care‐seeking patterns. The latter may in turn be explained by differences in the outcome of the fall but also in inclination to seek care and preferences in type of care (e.g. primary care vs. specialist care). The increased risk among people with ID of having at least one fall recorded during the study period could therefore be a result of a higher prevalence of falls, a higher risk of injury after falls or a higher likelihood of seeking specialist care when injured. Most likely, it is a combination of these three.

With respect to the first, prevalence of falls, previous studies investigating actual falls rather than health care visits due to falls have found an increased risk among older people with ID (Smulders *et al*. 2013). There may be several reasons for this. During the entire lifespan, people with ID are consistently found to have lower balance and gait capacities (Enkelaar *et al*. 2012), as well as poor perceptual‐motor coordination (Carmeli *et al*. 2008; Boot *et al*. 2013). They are more likely to have medical conditions known to affect fall risk, such as epilepsy (Lukaszyk *et al*. 2016; Cooper *et al*. 2017), diabetes mellitus (Vinik *et al*. 2017) and dementia (Axmon *et al*. 2016; Meuleners and Hobday 2017), as well as to be prescribed drugs known to increase the risk of falls, such as antidepressants, anxiolytics and hypnotics (Enderlin *et al*. 2015). Thus, potential ways of decreasing the prevalence of falls among older people with ID may be physical activity and physiotherapeutic interventions (Bartlo & Klein 2011; Van Hanegem *et al*. 2014; Hale *et al*. 2016) and regular drug reviews. A range of fall risk assessment tools has been found suitable for use among people with ID (Waninge *et al*. 2011a; Waninge *et al*. 2011b; Hilgenkamp *et al*. 2012; Enkelaar *et al*. 2013b; Salb *et al*. 2015). These include walking tests and tests of balance, mobility, physical fitness, gait capacities and strength. Performing such evaluations may also be a way to identify people with particularly high fall risk and take preventive measures among them. This, in turn, may decrease falls among older people with ID.

Among older people with ID, the majority of fall‐related injuries are minor, such as bruises, scratches and/or pain, and only about 10% constitute severe injuries (whereof a third were fractures) (Enkelaar *et al*. 2013a). This is similar to the 5–10% of falls resulting in fractures found in the older general population (Peeters *et al*. 2009). However, as falls are more common among people with ID, this translates into more fall‐related injuries in this population, which is also indicated by the results in the present study. We found that older people with ID had a different pattern of injuries than the general population in that they were more likely to injure their head and legs but less likely to injure their arms. High rates of face/head injuries among people with ID after a fall have also been found among adults (18+ years old) with ID (Grant *et al*. 2001; Cox *et al*. 2010; Geijer *et al*. 2014). A possible explanation for these type of injuries may be that people with ID fail to use their arms and hands to protect themselves when falling, again due to, e.g. difficulties integrating perceptual and visual information into motor action (Carmeli *et al*. 2008; Boot *et al*. 2013). It is important to be aware of this injury pattern when planning preventive measures to reduce fall‐related injuries among people with ID.

We have not been able to find any previously published data regarding differences between people with and without ID in inclination to seek specialist care after a fall. However, we know that older people with ID have a different health care utilisation pattern in general than people without ID, with higher proportions of unplanned care and lower proportions of planned care, especially in high ages (Sandberg *et al*. 2016). Whether such differences exist for fall‐induced health care can only be speculated on. People with ID, especially those at older ages, are likely to live in special housing adapted to their needs and with staff available around the clock. Whether a fall results in a hospital visit is therefore likely to depend on the decision of the staff rather than the person with ID hisself or herself. This in turn is likely to depend on how educated the staff are regarding falls and fall‐related injuries but also the ability of the person with ID to communicate pain as well as on the visual appearance of the injury. Unless the fall results in a visible injury, such as an open wound, people with ID might not themselves understand the need for health care, neither is it obvious to the caregiver that health care is required. This may partly explain the different patterns regarding type of injury, with dislocation, sprain and strain, as well as other or unspecified injury being less common in the ID cohort. Thus, it is important that staff working with people with ID have knowledge not only of fall prevention and communication but also of fall‐related injuries in this group.

That older people with ID fall at home or at an institution is most likely not an indicator of these places carrying a particular risk of falls, but rather that people with ID have few opportunities to be anywhere else than where they live, or that when going other places they have assistance. The LSS act is intended to ensure equal conditions for a fulfilling life for people with ID in relation to the general population. If they fall at their place of residence because they seldom go anywhere else, the act is not working as it is supposed to. A similar reasoning could be applied to activity when falling. If people with ID fall during vital activity because they rarely take part in other activities, the LSS act may not be not working as intended. In a recent study, leaders of group homes and daily activity centres highlighted the issue of decreased activity after retirement, suggesting that potential explanations were reduced social networks and lack of staff (Johansson *et al*. 2017). Further studies should investigate if LSS indeed provides enough support for people with ID to lead an active and safe life.

People with ID have more inpatient care and longer length of stay associated with falls. One reason for this could be that they have different injuries. If so, our results indicate that the fall‐related injuries sustained by people with ID are more severe than the injuries in the general population. Another explanation for the differences in health care utilisation could be that people with ID have more health problems and diagnoses (Sandberg *et al*. 2017) in addition to the fall itself, which could make the hospital stay more complicated. This could in turn be a reason for the higher degree of readmission seen among people with ID. It is important to investigate this further, to make sure that people with ID receive the care that they need after falls and are not discharged too early.

## Conclusions

When planning preventive measures to reduce falls and fall‐related injuries among older people with ID, it is important to acknowledge that they display a different pattern regarding place of and activity during the fall, as well as regarding the resulting injury than older people in the general population. Future studies should focus on risk factors for falls, fractures and other fall‐related injuries within the group of older people with ID.

## Conflict of Interest

None declared.

## References

[jir12564-bib-0001] Axmon A. , Karlsson B. & Ahlström G. (2016) Health care utilisation among older persons with intellectual disability and dementia: a registry study. Journal of Intellectual Disability Research 60, 1165–1177. 27730719 10.1111/jir.12338

[jir12564-bib-0002] Bartlo P. & Klein P. J. (2011) Physical activity benefits and needs in adults with intellectual disabilities: systematic review of the literature. American Journal on Intellectual and Developmental Disabilities 116, 220–232. 21591845 10.1352/1944-7558-116.3.220

[jir12564-bib-0003] Bigby C. (2007) Aging with and intellectual disability In: Comprehensive Guide to Intellectual & Developmental Disabilities (eds Brown I. & Percy M.), pp. 607–616. Brookes Publishing Co, Baltimore, MA.

[jir12564-bib-0004] Blackman N. (2007) People with learning disabilities – an ageing population. The Journal of Adult Protection 9, 3–8.

[jir12564-bib-0005] Boot F. H. , Pel J. J. , Vermaak M. P. , van der Steen J. & Evenhuis H. M. (2013) Delayed visual orienting responses in children with developmental and/or intellectual disabilities. Journal of Intellectual Disability Research 57, 1093–1103. 22974197 10.1111/j.1365-2788.2012.01610.x

[jir12564-bib-0006] Cahill S. , Stancliffe R. J. , Clemson L. & Durvasula S. (2014) Reconstructing the fall: individual, behavioural and contextual factors associated with falls in individuals with intellectual disability. Journal of Intellectual Disability Research 58, 321–332. 23373456 10.1111/jir.12015

[jir12564-bib-0007] Carmeli E. , Bar‐Yossef T. , Ariav C. , Levy R. & Liebermann D. G. (2008) Perceptual‐motor coordination in persons with mild intellectual disability. Disability and Rehabilitation 30, 323–329. 17852209 10.1080/09638280701265398

[jir12564-bib-0008] Cooper S.‐A. , Hughes‐McCormack L. , Greenlaw N. , McConnachie A. , Allan L. , Baltzer M. *et al* (2017) Management and prevalence of long‐term conditions in primary health care for adults with intellectual disabilities compared with the general population: a population‐based cohort study. Journal of Applied Research in Intellectual Disabilities Suppl 1, 68–81. 10.1111/jar.12386 28730746

[jir12564-bib-0009] Coppus A. (2013) People with intellectual disability: what do we know about adulthood and life expectancy? Developmental Disabilities Research Reviews 18, 6–16. 23949824 10.1002/ddrr.1123

[jir12564-bib-0010] Cox C. R. , Clemson L. , Stancliffe R. J. , Durvasula S. & Sherrington C. (2010) Incidence of and risk factors for falls among adults with an intellectual disability. Journal of Intellectual Disability Research 54, 1045–1057. 21105935 10.1111/j.1365-2788.2010.01333.x

[jir12564-bib-0011] Enderlin C. , Rooker J. , Ball S. , Hippensteel D. , Alderman J. , Fisher S. J. *et al* (2015) Summary of factors contributing to falls in older adults and nursing implications. Geriatric Nursing 36, 397–406. 26343008 10.1016/j.gerinurse.2015.08.006

[jir12564-bib-0012] Enkelaar L. , Smulders E. , Lantman‐de Valk H. V. , Weerdesteyn V. & Geurts A. C. H. (2013a) Prospective study on risk factors for falling in elderly persons with mild to moderate intellectual disabilities. Research in Developmental Disabilities 34, 3754–3765. 24029799 10.1016/j.ridd.2013.07.041

[jir12564-bib-0013] Enkelaar L. , Smulders E. , van Schrojenstein Lantman‐de Valk H. , Geurts A. C. & Weerdesteyn V. (2012) A review of balance and gait capacities in relation to falls in persons with intellectual disability. Research in Developmental Disabilities 33, 291–306. 22018534 10.1016/j.ridd.2011.08.028

[jir12564-bib-0014] Enkelaar L. , Smulders E. , van Schrojenstein Lantman‐de Valk H. , Weerdesteyn V. & Geurts A. C. (2013b) Clinical measures are feasible and sensitive to assess balance and gait capacities in older persons with mild to moderate intellectual disabilities. Research in Developmental Disabilities 34, 276–285. 22985782 10.1016/j.ridd.2012.08.014

[jir12564-bib-0015] Evenhuis H. M. , Hermans H. , Hilgenkamp T. I. , Bastiaanse L. P. & Echteld M. A. (2012) Frailty and disability in older adults with intellectual disabilities: results from the healthy ageing and intellectual disability study. Journal of the American Geriatrics Society 60, 934–938. 22587856 10.1111/j.1532-5415.2012.03925.x

[jir12564-bib-0016] Finlayson J. (2018) Fall prevention for people with learning disabilities: key points and recommendations for practitioners and researchers. Tizard Learning Disability Review 23, 91–99.

[jir12564-bib-0017] Finlayson J. , Morrison J. , Jackson A. , Mantry D. & Cooper S. A. (2010) Injuries, falls and accidents among adults with intellectual disabilities. Prospective cohort study. Journal of Intellectual Disability Research 54, 966–980. 21040056 10.1111/j.1365-2788.2010.01319.x

[jir12564-bib-0018] Foran S. , McCallion P. & McCarron M. (2016) The prevalence of falls among older adults with intellectual disability in Ireland. Age and Ageing 45, 55.

[jir12564-bib-0019] Geijer J. R. , Stanish H. I. , Draheim C. C. & Dengel D. R. (2014) Bone mineral density in adults with Down syndrome, intellectual disability, and nondisabled adults. American Journal on Intellectual and Developmental Disabilities 119, 107–114. 24679348 10.1352/1944-7558-119.2.107

[jir12564-bib-0020] Grant H. , Pickett W. , Lam M. , O'Connor M. & Ouellette‐Kuntz H. (2001) Falls among persons who have developmental disabilities in institutional and group home settings. Journal on Developmental Disabilities 8, 57–73.

[jir12564-bib-0021] Hale L. A. , Mirfin‐Veitch B. F. & Treharne G. J. (2016) Prevention of falls for adults with intellectual disability (PROFAID): a feasibility study. Disability and Rehabilitation 38, 36–44. 25714511 10.3109/09638288.2015.1017613

[jir12564-bib-0022] Haveman M. , Heller T. , Lee L. , Maaskant M. , Shooshtari S. & Strydom A. (2010) Major health risks in aging persons with intellectual disabilities: an overview of recent studies. Journal of Policy and Practice in Intellectual Disabilities 7, 59–69.

[jir12564-bib-0023] Haveman M. , Perry J. , Salvador‐Carulla L. , Walsh P. N. , Kerr M. , Lantman‐de Valk H. V. *et al* (2011) Ageing and health status in adults with intellectual disabilities: results of the European POMONA II study. Journal of Intellectual & Developmental Disability 36, 49–60. 21314593 10.3109/13668250.2010.549464

[jir12564-bib-0024] Hilgenkamp T. I. M. , van Wijck R. & Evenhuis H. M. (2012) Feasibility and reliability of physical fitness tests in older adults with intellectual disability: a pilot study. Journal of Intellectual and Developmental Disability 37, 158–162. 22545938 10.3109/13668250.2012.681773

[jir12564-bib-0025] Hsieh K. , Heller T. & Miller A. B. (2001) Risk factors for injuries and falls among adults with developmental disabilities. Journal of Intellectual Disability Research 45, 76–82. 11168779 10.1046/j.1365-2788.2001.00277.x

[jir12564-bib-0026] Hsieh K. , Rimmer J. & Heller T. (2012) Prevalence of falls and risk factors in adults with intellectual disability. Ajidd‐American Journal on Intellectual and Developmental Disabilities 117, 442–454. 10.1352/1944-7558-117.6.442 23167484

[jir12564-bib-0027] Janicki M. P. , Dalton A. J. , Henderson C. M. & Davidson P. W. (1999) Mortality and morbidity among older adults with intellectual disability: health services considerations. Disability and Rehabilitation 21, 284–294. 10381241 10.1080/096382899297710

[jir12564-bib-0028] Johansson M. , Björne P. , Runesson I. & Ahlström G. (2017) Healthy ageing in people with intellectual disabilities from managers' perspective: a qualitative study. Healthcare (Basel) 5, pii: E45. 10.3390/healthcare5030045 PMC5618173 28820435

[jir12564-bib-0029] Kapell D. , Nightingale B. , Rodriguez A. , Lee J. H. , Zigman W. B. & Schupf N. (1998) Prevalence of chronic medical conditions in adults with mental retardation: comparison with the general population. Mental Retardation 36, 269–279. 9713183 10.1352/0047-6765(1998)036<0269:POCMCI>2.0.CO;2

[jir12564-bib-0030] Lukaszyk C. , Harvey L. , Sherrington C. , Keay L. , Tiedemann A. , Coombes J. *et al* (2016) Risk factors, incidence, consequences and prevention strategies for falls and fall‐injury within older indigenous populations: a systematic review. Australian and New Zealand Journal of Public Health 40, 564–568. 27774702 10.1111/1753-6405.12585

[jir12564-bib-0031] Meuleners L. B. & Hobday M. B. (2017) A population‐based study examining injury in older adults with and without dementia. Journal of the American Geriatrics Society 65, 520–525. 28102889 10.1111/jgs.14523

[jir12564-bib-0032] Pal J. , Hale L. , Mirfin‐Veitch B. & Claydon L. (2014) Injuries and falls among adults with intellectual disability: a prospective New Zealand cohort study. Journal of Intellectual and Developmental Disability 39, 35–44.

[jir12564-bib-0033] Peeters G. , van Schoor N. M. & Lips P. (2009) Fall risk: the clinical relevance of falls and how to integrate fall risk with fracture risk. Best Practice & Research. Clinical Rheumatology 23, 797–804. 19945691 10.1016/j.berh.2009.09.004

[jir12564-bib-0034] Salb J. , Finlayson J. , Almutaseb S. , Scharfenberg B. , Becker C. , Sieber C. *et al* (2015) Test‐retest reliability and agreement of physical fall risk assessment tools in adults with intellectual disabilities. Journal of Intellectual Disability Research 59, 1121–1129. 26294089 10.1111/jir.12216

[jir12564-bib-0035] Sandberg M. , Ahlström G. , Axmon A. & Kristensson J. (2016) Somatic healthcare utilisation patterns among older people with intellectual disability: an 11‐year register study. BMC Health Services Research 16, 642. 27829424 10.1186/s12913-016-1880-x PMC5103402

[jir12564-bib-0036] Sandberg M. , Ahlström G. & Kristensson J. (2017) Patterns of somatic diagnoses in older people with intellectual disability: a Swedish eleven year case‐control study of inpatient data. Journal of Applied Research in Intellectual Disabilities 30, 157–171. 26542759 10.1111/jar.12230

[jir12564-bib-0037] SFS 1993:387 (1993) Act concerning support and service for persons with certain functional impairments [In Swedish: Lag om stöd och service till vissa funktionshindrade]. Stockholm, Sweden.

[jir12564-bib-0038] Sherrard J. , Tonge B. J. & Ozanne‐Smith J. (2001) Injury in young people with intellectual disability: descriptive epidemiology. Injury Prevention 7, 56–61. 11289537 10.1136/ip.7.1.56 PMC1730696

[jir12564-bib-0039] Skorpen S. , Nicolaisen M. & Langballe E. M. (2016) Hospitalisation in adults with intellectual disabilities compared with the general population in Norway. Journal of Intellectual Disability Research 60, 365–377. 26915087 10.1111/jir.12255

[jir12564-bib-0040] Smulders E. , Enkelaar L. , Weerdesteyn V. , Geurts A. C. H. & Valk H. (2013) Falls in older persons with intellectual disabilities: fall rate, circumstances and consequences. Journal of Intellectual Disability Research 57, 1173–1182. 23106830 10.1111/j.1365-2788.2012.01643.x

[jir12564-bib-0041] Van Hanegem E. , Enkelaar L. , Smulders E. & Weerdesteyn V. (2014) Obstacle course training can improve mobility and prevent falls in people with intellectual disabilities. Journal of Intellectual Disability Research 58, 485–492. 23600491 10.1111/jir.12045

[jir12564-bib-0042] Wang D. , McDermott S. & Sease T. (2002) Analysis of hospital use for injury among individuals with mental retardation. Injury Control and Safety Promotion 9, 107–111. 12461837 10.1076/icsp.9.2.107.8703

[jir12564-bib-0043] Waninge A. , Evenhuis I. J. , van Wijck R. & van der Schans C. P. (2011a) Feasibility and reliability of two different walking tests in people with severe intellectual and sensory disabilities. Journal of Applied Research in Intellectual Disabilities 24, 518–527.

[jir12564-bib-0044] Waninge A. , van Wijck R. , Steenbergen B. & van der Schans C. P. (2011b) Feasibility and reliability of the modified Berg Balance Scale in persons with severe intellectual and visual disabilities. Journal of Intellectual Disability Research 55, 292–301. 21155916 10.1111/j.1365-2788.2010.01358.x

[jir12564-bib-0045] World Health Organization (WHO) (2007) WHO Global Report on Falls Prevention in Older Age. WHO, France.

[jir12564-bib-0046] Vinik A. I. , Camacho P. , Reddy S. , Valencia W. M. , Trence D. , Matsumoto A. M. *et al* (2017) Aging, diabetes, and falls. Endocrine Practice 23, 1117–1139. 28704101 10.4158/EP171794.RA

